# Meeting of the Commissioners on the Location of the Insane Asylum

**Published:** 1869-12

**Authors:** 


### Meeting of the Commissioners on the Location of the Insane Asylum.

The Board of Commissoners appointed by Gov. Hoffman to locate the new Insane Asylum. met in this city Dec. 22d, and unanimously confirmed. in their report to the Governor. their previous decision, which selected Buffalo as its most desirable and appropriate location. The city offer the site, the cost of which is not more than $50,000, and a perpetual gratuitous supply of water from the city mains. Dr. Gray, of Utica, the chairman of the board, while in town visited the Medical College, and, in addressing the students, said that he was strongly confident that the method of clinical instruction in mental diseases and insanity just undertaken, in New York city, by Prof. Hammond, would prove quite successful, and be of great practical benefit. He anticipated for the students the time when such instruction should be given in Buffalo with the superior facilities which will then be afforded.

